# The Prognostic Implication of Left Atrial Strain Parameters with Conventional Left Atrial Parameters for the Prediction of Adverse Outcomes in Asian Patients with Hypertrophic Cardiomyopathy—An Echocardiographic Study

**DOI:** 10.3390/jcdd12070261

**Published:** 2025-07-08

**Authors:** Andre Seah, Tony Y. W. Li, Novi Yanti Sari, Chi-Hang Lee, Tiong-Cheng Yeo, James W. L. Yip, Yoke Ching Lim, Kian-Keong Poh, William K. F. Kong, Weiqin Lin, Ching-Hui Sia, Raymond C. C. Wong

**Affiliations:** 1 Department of Medicine, National University Health System, Singapore 119074, Singapore; andre.seah@mohh.com.sg; 2 Department of Cardiology, National University Heart Centre, Singapore 119228, Singapore; tony_li@nuhs.edu.sg (T.Y.W.L.); mdclchr@nus.edu.sg (C.-H.L.); tiong_cheng_yeo@nuhs.edu.sg (T.-C.Y.); james_yip@nuhs.edu.sg (J.W.L.Y.); yoke_ching_lim@nuhs.edu.sg (Y.C.L.); mdcpkk@nus.edu.sg (K.-K.P.); william_kong@nuhs.edu.sg (W.K.F.K.); weiqin_lin@nuhs.edu.sg (W.L.); raymond_cc_wong@nuhs.edu.sg (R.C.C.W.); 3 Department of Cardiology, Dr. Muhammad Hoesin General Hospital, Palembang 30126, Indonesia; novitham.md@gmail.com; 4 Yong Loo Lin School of Medicine, National University of Singapore, Singapore 117549, Singapore

**Keywords:** hypertrophic cardiomyopathy, heart failure, left atrial strain

## Abstract

Background/Objectives: Left atrial function can be a tool for risk stratification for hypertrophic cardiomyopathy (HCM). Over the past decade, there has been growing interest in the application of strain analysis for earlier and more accurate prediction of cardiovascular disease prognosis. This study aimed to investigate the performance of left atrial strain analysis compared to conventional left atrial measures in predicting clinical outcomes in Asian patients with HCM. Methods and Results: This was a retrospective study involving 291 patients diagnosed with HCM between 2010 and 2017. Left atrial volumes were assessed using the method of discs in orthogonal plans at both end diastole and end systole. Left atrial (LA) strain was obtained using a post-hoc analysis with TOMTEC software. We tested the various left atrial parameters against outcomes of (1) heart failure hospitalization and (2) event-free survival from a composite of adverse events, including all-cause mortality, ventricular tachycardia (VT)/ventricular fibrillation (VF) events, appropriate device therapy if an implantable cardioverter defibrillator (ICD) was implanted, stroke, and heart failure hospitalization. The patients had a mean age of 59.0 ± 16.7 years with a male preponderance (71.2%). The cumulative event-free survival over a follow-up of 3.9 ± 2.7 years was 55.2% for patients with an abnormal LA strain versus 82.4% for patients without one (*p* < 0.001). Multivariable Cox regression analyses were performed separately for each LA parameter, adjusting for age, sex, LV mass index, LV ejection fraction (EF), E/e’, the presence of LV outflow tract (LVOT) obstruction at rest, and atrial fibrillation. An analysis showed that all parameters except for LAEF demonstrated an independent association with heart failure hospitalization. Left atrial strain outperformed the rest of the parameters by demonstrating an association with a composite of adverse events. Conclusions: In Asian patients with HCM, measures of left atrial strain were independently associated with heart failure hospitalization and a composite of adverse outcomes. Left atrial strain may be used as a tool to predict adverse outcomes in patients with HCM.

## 1. Introduction

Hypertrophic cardiomyopathy (HCM) is the most common inherited genetic cardiomyopathy [1]. Heart failure is an important complication of HCM with high mortality rates and less recognized direct therapeutic options [2]. This is in contrast with sudden cardiac death, for which there are established risk stratification scores and therapeutic options, e.g., implantable cardioverter defibrillator (ICD) placement. The left ventricular ejection fraction (LVEF) represents the most used metric for the evaluation of systolic function in patients with heart failure. However, it is known to have poor sensitivity in the identification of LV systolic dysfunction in patients with HCM as it decreases only in the later stages [3]. Left ventricular (LV) global longitudinal strain (GLS) is increasingly recognized as a tool to detect early LV systolic dysfunction [4]. LV-GLS is an example of strain echocardiography and measures the degree of deformation of the myocardium during the contraction phase of the cardiac cycle.

In broader populations, progressive enlargement and remodeling of the left atrium have been predictive of the development of heart failure, especially in heart failure with preserved ejection fraction (HFpEF) [5,6]. These changes are expected in HCM as well, purportedly due to progressive hypertrophy of the LV with elevation in both LA and LV filling pressures [7,8,9,10]. Given that LV-GLS has been established to be a useful marker in the management of HCM [11], the authors postulated that measures of LA strain may likewise allow for earlier detection of LA remodeling. Left atrial indices have been shown to be associated with the development of atrial fibrillation, and atrial fibrillation has been shown to be associated with heart failure hospitalizations in patients with HCM [6,12,13,14]. We therefore aimed to examine whether LA strain, as a measure of LA remodeling, was associated with the development of heart failure and adverse outcomes in HCM.

## 2. Materials and Methods

### 2.1. Study Population

This study included patients diagnosed with HCM between 2011 and 2017 at a single tertiary academic medical center, identified retrospectively from an echocardiography database maintained at the center. Subjects with reported left ventricular hypertrophy or hypertrophic cardiomyopathy on the indication or diagnosis of transthoracic echocardiograms performed between 2011 to 2017 were identified, and their charts were reviewed; patients for whom a diagnosis of hypertrophic cardiomyopathy was made by a cardiologist were selected. This yielded a final cohort of 291 subjects following the exclusion of 4 patients with suboptimal TTE images. HCM was diagnosed in a patient based on clinical and echocardiographic features in accordance with prevailing guidelines, which required the presence of myocardial hypertrophy in the absence of local or systemic etiologies with loading conditions [15]. Ethics approval was obtained from the National Healthcare Group Domain Specific Review Board (reference number: 2021/00623).

### 2.2. Echocardiographic Analysis

Echocardiographic data was obtained from the index echocardiogram. Basic echocardiographic data was interpreted and analyzed according to standard protocols based on the American Society of Echocardiography guidelines [16]. Conventional echocardiographic parameters including left ventricular ejection fraction (LVEF), left ventricular mass index (LVMI), maximal wall thickness, LV outflow tract (LVOT) gradient, the presence and severity of mitral regurgitation (MR), and LV E/e’ were captured. Additional baseline demographics and clinical data were retrospectively obtained at the point of the index echocardiogram.

### 2.3. Left Atrial Parameters

Left atrial volumetric assessments were made retrospectively. The LA volume was measured in the four-chamber and two-chamber apical views by tracing the LA inner border, excluding atrial appendage and pulmonary veins, and then calculated using the disk summation technique. The maximum (LAVImax) and minimum LA volumes (LAVImin) were measured at end systole and end diastole, respectively, and subsequently indexed for body surface area. LAEF is calculated as follows:

$$LAEF=\frac{LAmax-LAmin}{LAmax}\times 100,$$

and is expressed as a percentage. A single cardiologist measured all parameters and determined strain.

### 2.4. Strain Analysis

**Figure 1:**
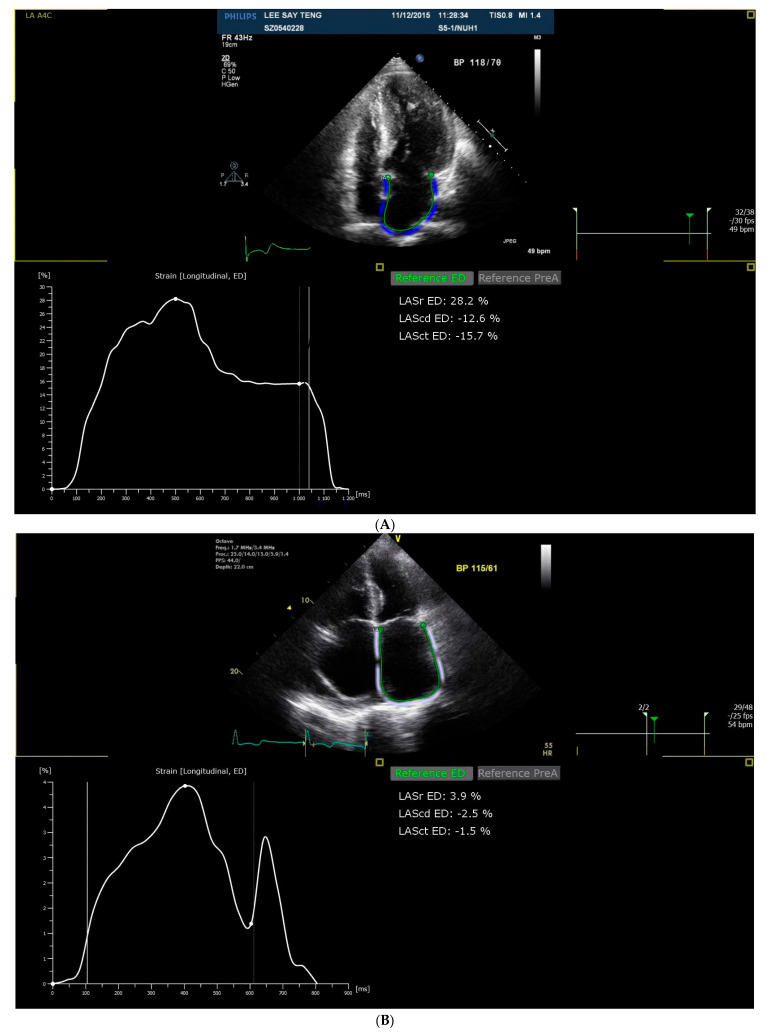
(**A**). Measurement of LA strain in a patient with good LA strain. (**B**). Measurement of LA strain in a patient with poor LA strain.

Strain analysis was performed post-hoc based on the non-foreshortened echocardiographic images in the apical 4-, 3-, and 2-chamber views using a commercial software, TomTec-Arena version 4.6 (TOMTEC Imaging Systems GmbH, Munich, Germany). This allowed for the generation of reservoir (LASr), conduit (LAScd), and contraction strain (LASct). The LA contours were initially automatically traced using the TomTec software and manually checked for accuracy and adjusted accordingly. The patients were classified as normal or abnormal strain based on accepted reference values from the literature, where cut-offs of 23%, 12%, and 5% were adopted for LASr, LAScd, and LASct strain, respectively [17,18]. Figure 1A,B demonstrate the measurement of LA strain in patients with good LA strain and poor LA strain, respectively.

The LA endocardial border is traced in the apical 4-chamber view, and LA strain is measured at the reservoir, conduit, and contraction phases.

### 2.5. Follow-Up and Clinical Endpoints

The patients were followed for a mean of 3.9 ± 2.7 years. Outcome data were collected until 31 December 2021 based on a review of the hospital’s medical records. Outcomes collected included first admission for heart failure as well as all-cause mortality. Mortality data in our hospital records are synchronized to the national death registry such that we were able to capture all mortality events. Medical records were also screened for records of implantation of an ICD. If an ICD was implanted, the device interrogation records were screened to adjudicate for records of device therapy. We finally assessed our cohort for outcomes of (1) heart failure hospitalization and (2) event-free survival from a composite of adverse events, including all-cause mortality, VT/VF events, appropriate device therapy if an ICD was implanted, stroke, and heart failure hospitalization.

### 2.6. Statistical Analysis

For left atrial volumetric parameters, we adopted the widely accepted cut-off of >34 mL/m^2^ for abnormal LAVImax values [16]. ROC analysis was performed to derive the cut-off for LAVImin and LAEF. For LA strain, we adopted routine cut-offs of 23%, 12%, and 5% for LASr, LAScd, and LASct strain, respectively, based on the literature [19].

Continuous variables were expressed as the mean (± standard deviation), while categorical variables were expressed as a number (proportion). *p*-values were 2-sided and deemed significant if <0.05. Cumulative event-free survival rates for the overall population, stratified by the various left atrial parameters, were calculated using the Kaplan–Meier method. The log-rank test was used to compare the above-defined groups. Multivariable Cox regression analyses were performed separately for each LA parameter, adjusting for age, sex, LV mass index, LV ejection fraction, E/e’, the presence of LVOT obstruction at rest, and atrial fibrillation. The concordance statistic (C-statistic) was calculated for each of the multivariate models to determine its discriminatory power. A statistical analysis was performed using SPSS 27 [20] and MedCalc Statistical Software version 19.2.6 [21].

## 3. Results

The present study comprised 291 patients with HCM, following the exclusion of 4 patients for whom their index echocardiogram was of suboptimal image quality. Baseline demographic, clinical, and echocardiographic data are presented below in Table 1.

The mean age was 59.0 ± 16.7 years, with male preponderance (71.2%, *n* = 207). The cohort consisted of patients of Chinese (65.3%, *n* = 190), Malay (18.6%, *n* = 54), and Indian (12.4%, *n* = 36) descent. The apical subtype of HCM was found in 36.1% of patients (*n* = 105), which represented a larger proportion than previously reported in Asian cohorts [22]. Patients who developed heart failure were predominantly male, of older age, and had more comorbidities like hypertension, diabetes mellitus, and ischemic heart disease, similar to the cohort of patients who experienced the composite adverse outcome. During the mean follow-up of 3.9 ± 2.7 years, 34 patients were admitted for heart failure, while 101 patients developed adverse events including stroke, heart failure hospitalization, a sudden cardiac arrest event, or mortality.

### 3.1. Univariate Analysis

The various demographic, clinical, and echocardiographic parameters were assessed for their relation to the development of heart failure and composite adverse outcomes, and the results are shown in Table 1 and Table 2, respectively.

The LV ejection fraction, LV mass index, LV E/e’, and the presence of more than moderate mitral regurgitation were all associated with heart failure hospitalization. Conventional volumetric LA parameters and LA strain metrics were all found to be independently associated with heart failure hospitalization; likewise, these observations were made for composite adverse outcomes.

### 3.2. LA Strain

**Figure 2:**
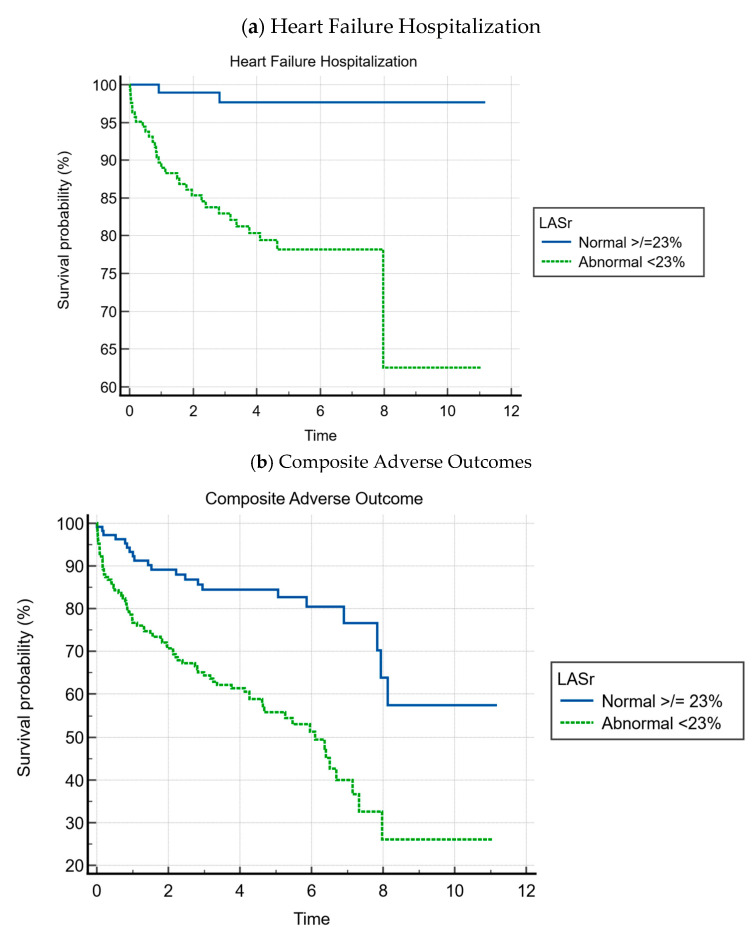
Kaplan–Meier curves for LASr ED.

For LA strain, we decided to adopt cut-offs that have been reported in the literature for our analysis. This included cut-offs of 23%, 12%, and 5% for LASr, LAScd, and LASct strain, respectively [18]. Utilizing each of these cut-offs, all three parameters were significantly associated with both heart failure hospitalization and the composite of adverse outcomes. A time-to-event analysis was also conducted for LA reservoir strain, with abnormal strain being defined as LASr ED ≥23%, which is shown in Figure 2.

The cumulative event-free survival over a follow-up average of 3.9 ± 2.7 years was as follows:

- Heart failure event-free survival was 81.8% for patients with abnormal LASr vs. 98.3% for patients with normal LASr (*p* < 0.001);
- Composite event-free survival was 55.2% for patients with abnormal LASr vs. 82.4% for patients with normal LASr (*p* < 0.001).

**Figure 3:**
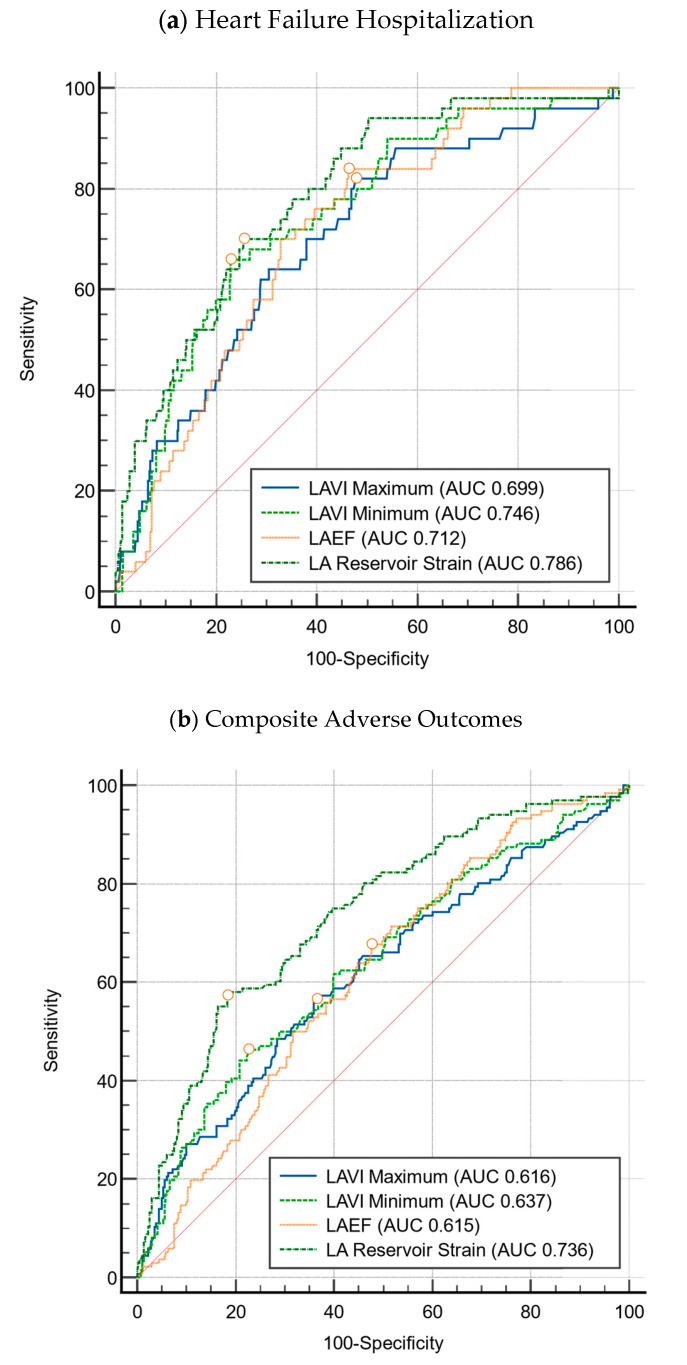
Comparison of left atrial parameters for HCM. Circles represent the cut-offs of the left atrial indices.

**Figure A1:**
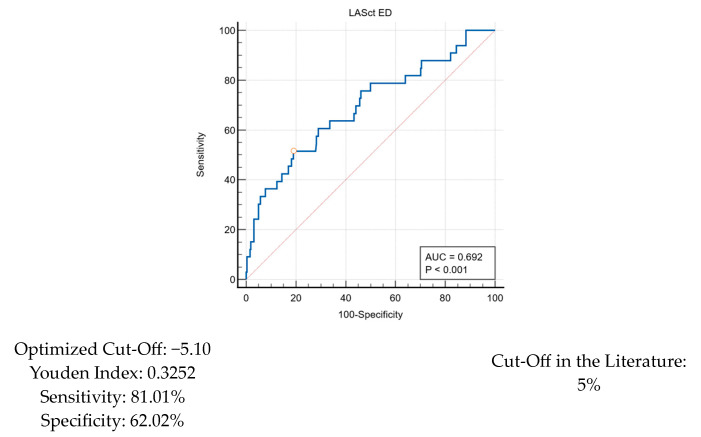

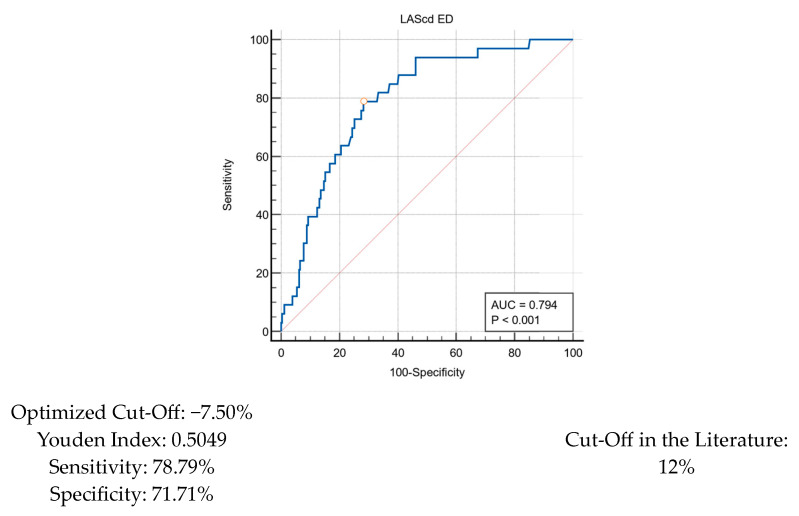
ROC curve for LA parameters.

Heart failure event-free survival was 81.8% for patients with abnormal LA reservoir strain compared to 98.3% for patients with normal LA reservoir strain (*p* < 0.001). This relationship was also found in terms of composite adverse outcomes, wherein composite event-free survival was 55.2% for patients with abnormal LA reservoir strain compared to 82.4% for patients with normal LA reservoir strain (*p* < 0.001). The predictive power of the LA strain parameters, LASr, LAScd, and LASct, were also corroborated by the ROC curve analysis for which the AUC was 0.786, 0.794, and 0.692, respectively, as shown in Figure 3 and Figure A1.

### 3.3. Multivariate Cox Regression Analysis

Multivariate Cox regression analyses, adjusting for age, sex, LV mass index, LV ejection fraction, the presence of resting LV outflow tract obstruction, LV E/e’, and atrial fibrillation, were performed separately for all LA parameters studied, and the results are shown in Table 3. This includes LA volumetric analysis with the LAVImax and LAVImin, LA ejection fraction, and LA strain analysis.

All parameters, except for LAEF (*p* = 0.053), were found to be independently associated with heart failure hospitalization, with an incremental value in predicting future heart failure hospitalizations compared to the baseline multivariate model, as demonstrated by the likelihood ratio ꭓ2 test. In testing for the composite adverse out-come, LASr strain (HR 2.01, 95% CI [01.56–3.481]), LAScd strain (HR 3.31, 95% CI [1.84–6.97]), and LASct strain (HR 2.47, 95% CI [1.57–3.88]) demonstrated incremental values in the prediction of composite adverse outcomes, while the addition of LAVImin or LAVImax or LAEF did not improve the risk discrimination of the model.

## 4. Discussion

This study demonstrated the association of various indicators of left atrial remodeling with future heart failure hospitalization in patients with HCM. Measures of LA strain outperformed conventional LA parameters in stratifying risk for heart failure hospitalization or a composite of adverse outcomes. Heart failure remains an area of unmet need in the care of patients with HCM, with the most used measure of heart function, LVEF, only decreasing late in disease. LA strain may allow for the earlier identification of patients with a predisposition for heart failure.

The association between left atrial structural and functional remodeling and AF development in HCM has been established by previous studies [23,24], which predisposes individuals to the development of heart failure [25]. LA remodeling therefore precedes the development of heart failure. This is demonstrated by the current study, which shows the association of LA remodeling with heart failure, regardless of the presence of AF.

An increase in LA diameter is known to be associated with the development of AF in HCM [24]. LA volume, most commonly assessed as LAVImax, has been shown to be a more accurate representation of LA remodeling than the LA anteroposterior diameter, being more predictive of AF or cardiovascular events in general [26]. Subsequent to the growing acceptance of LAVImax, LAVImin was then reported to possibly be an even more accurate assessment of LA remodeling [27] as the LA is more directly exposed to LV pressures during end diastole compared to end systole wherein the systolic displacement of the mitral annular plane toward the LV apex stretches the LA and distorts the LAVImax. This study supports the use of LAVImin in predicting heart failure hospitalization and composite adverse outcomes in patients with HCM.

There has been growing interest to assess LA function in LA strain. Like the use of LVGLS in detecting left ventricular systolic dysfunction, LA strain has the potential to pick up LA dysfunction earlier as well [28]. Previous studies have examined LA strain in a general heart failure population [29] and an HCM population [30,31]. Lee et al. demonstrated the improved predictive power of LA reservoir strain in heart failure outcomes in the HCM population over conventional volumetric measurements such as LA diameter and LAVI [14]. This study validated that association in a multi-ethnic Asian population and extended the analysis to study the predictive power of a composite of clinically relevant adverse outcomes. These studies have consistently shown the predictive power of LA strain parameters in prognosticating heart failure and adverse events. Our study similarly demonstrates that LA strain in all three components (reservoir, conduit, or contractile strain) can demonstrate superior risk stratification power to LA volumetric parameters. LA strain parameters may capture increased LV filling pressures prior to LA enlargement and hence be more sensitive to changes in LV filling pressures [32,33,34]. This can be a useful way to further risk stratify patients with HCM in the future.

### 4.1. Clinical Implications

Currently, there are few established effective and specific pharmacological therapeutic options for patients with HCM, especially in apical and non-obstructive HCM, which are more common in the Asian demographic. Mavacamten, the first-in-class drug that has provided disease specific therapy for symptomatic patients with obstructive HCM, improved symptoms and decreased the need for septal reduction therapy in VALOR-HCM and improved exercise capacity, LVOT obstruction, and NYHA functional class in EXPLORER-HCM [23,33,35]. However, the recently concluded ODYSSEY-HCM trial, which studied mavacamten in non-obstructive HCM, was a negative trial [36].

In classical HCM, the LVOT gradient identifies patients with and without obstruction, with symptomatic obstructive patients potentially being eligible for disease-directed therapy. In contrast, no comparable, clinically applicable marker currently exists for apical or other non-obstructive HCM. A minority of patients with non-obstructive HCM experience progression of symptoms to NYHA Class III/IV, even with preserved ejection fraction [37], with most HCM patients having hyperdynamic or normal LVEF. The LVEF is thus insufficient in the assessment and prognostication of non-obstructive HCM patients.

This study demonstrated that LA strain provides incremental prognostic value over the LVEF and volumetric LA parameters and may serve as a more sensitive marker in the general HCM population, complementing the LVOT gradient for patients of obstructive phenotype. Further studies needs to be conducted, specifically in the non-obstructive subgroup of patients, to validate its utility. A prognostic marker able to classify non-obstructive patients as those with good and poor outcomes would facilitate the practice of cost-effective and more clinically meaningful care for this diverse group of patients. Ultimately, more research into disease-directed therapy for non-obstructive HCM needs to be conducted for this underserved population.

Separately, LA strain may have a role in explaining previously unaccounted for sex differences in HCM. A recent study analyzing the clinical outcomes for patients with HCM stratified by sex found that women with HCM have a higher risk of progression to heart failure [38]. There was no difference in systolic function between sex to account for this higher risk, nor was it explained completely by more significant obstructive disease in the form of LVOT gradient at rest, leaving the authors to postulate that greater diastolic dysfunction may explain this preponderance. The addition of LA strain parameters to current markers of diastolic dysfunction, particularly conduit and contractile strain, may detect smaller differences in the degree of diastolic dysfunction between sex that may account for the higher propensity for heart failure seen in women with HCM [37]. Further research is needed in this respect to determine whether LA strain could be useful in detecting diastolic dysfunction earlier, especially for female patients, for whom delayed presentation with atypical symptoms of cardiovascular disease, resulting in delayed intervention, has often been described [39,40,41].

Finally, an interesting emerging role of strain analysis is in rapid risk stratification in the emergency room setting. In a prospective cohort of patients with acute ischemic stroke without atrial fibrillation, global left atrial strain of less than 20%, performed 6–12 h within hospital admissions, was found to be predictive of death and re-hospitalizations [42]. Battel et al. reported a case study of a patient with HCM and preserved ejection fraction who developed atrial fibrillation with CHA_2_DS_2_-VASc 2, and a decision was made to initiate anticoagulation prompted by a GLS value of −6.2%, which suggests stasis and hence predisposition for thrombus formation [41]. These findings highlight the clinical utility of strain analysis, even in the emergency room setting, as it can provide rapid and reliable prognostic information that may potentially influence clinical decision making [43,44]. This study lends further credence to the clinical value of the incremental prognostic value provided by LA strain analysis.

### 4.2. Limitations

There are a few limitations to this study. Firstly, due to the design of the study, outpatient presentations of heart failure were not recorded, and hence, only heart failure hospitalizations were captured. Another major limitation is that only all-cause mortality was reported. Due to limitations in the linkages of electronic health records nationally, cardiac mortality could not be specifically identified unless the patient passed away within our institution. Lastly, as a retrospective study, we could only establish association and not causality.

## 5. Conclusions

In conclusion, in Asian patients with HCM, measures of left atrial strain demonstrated incremental predictive value for heart failure hospitalization and a composite of adverse outcomes. Further studies with larger sample sizes are required to establish the plausible mechanistic process linking LA functional and volumetric parameters with clinical outcomes, particularly stroke and mortality.

## Figures and Tables

**Table 1:** Characteristics of HCM patients in relation to heart failure hospitalization.

Variables	Overall (*n* = 291)	Heart Failure Hospitalization (*n* = 33)	Free from Heart Failure (*n* = 258)	Hazard Ratio (95% CI)	*p*-Value
**Baseline Demographics**					
Age (years)	58.75 ± 16.79	70.58 ± 16.79	57.24 ± 16.68	1.06 (1.03–1.09)	<0.001
Female, n (%)	82 (28.2%)	19 (57.6%)	63 (24.4%)	4.20 (1.99–8.86)	<0.001
BMI	25.30 ± 4.48	24.59 ± 4.19	25.39 ± 4.52	0.96 (0.88–1.04)	0.330
Ethnicity				-	0.549
Chinese	194 (66.7%)	24 (72.7%)	170 (65.9%)		
Malay	53 (18.2%)	5 (15.2%)	48 (18.6%)		
Indian	35 (12.0%)	4 (12.1%)	31 (12.0%)		
Other	9 (3.1%)	0 (0.0%)	9 (3.5%)		
**Comorbidities**					
Hypertension	131 (45.0%)	22 (67.6%)	109 (42.2%)	2.73 (1.27–5.87)	0.009
Hyperlipidemia	117 (40.2%)	17 (51.5%)	100 (38.8%)	1.68 (0.81–3.47)	0.188
Diabetes Mellitus	47 (16.2%)	10 (30.3%)	37 (14.3%)	2.60 (1.14–5.90)	0.040
Ischemic Heart Disease	80 (27.5%)	16 (48.5%)	64 (24.8%)	2.85 (1.36–5.97)	0.007
Atrial Fibrillation	51 (17.5%)	11 (21.6%)	40 (15.5%)	2.73 (1.23–6.06)	0.025
Chronic Kidney Disease	3 (1.0%)	0 (0.0%)	3 (1.2%)	-	>0.999
Previous Stroke	31 (10.7%)	6 (18.2%)	25 (9.7%)	2.07 (0.78–5.50)	0.139
**Medication Use**					
Antiplatelet	87 (29.9%)	15 (45.5%)	72 (27.9%)	2.15 (1.03–4.50)	0.045
Oral Anticoagulation	58 (20.0%)	16 (48.5%)	42 (16.3%)	4.98 (2.33–10.65)	0.001
Beta Blockers	189 (64.9%)	27 (81.8%)	162 (62.8%)	2.67 (1.06–6.69)	0.033
ACE-I/ARB/ARNI	82 (28.2%)	14 (42.4%)	68 (26.4%)	2.06 (0.98–4.33)	0.065
Calcium Channel Blockers	58 (19.9%)	8 (24.2%)	50 (19.4%)	1.33 (0.57–3.13)	0.493
Diuretics	49 (16.8%)	15 (45.5%)	34 (13.2%)	5.49 (2.53–11.91)	<0.001
Oral Antiarrhythmics	17 (5.8%)	6 (18.2%)	11 (4.3%)	4.99 (1.71–14.56)	0.007
Type of HCM					
Sigmoid Septum	52 (17.9%)	7 (21.2%)	45 (17.4%)	-	0.610
Reverse Curvature	44 (15.1%)	2 (6.1%)	42 (16.3%)		
Apical	105 (36.1%)	14 (42.4%)	91 (35.3%)		
Concentric	85 (29.2%)	9 (27.3%)	76 (29.5%)		
Mid Cavity	1 (0.3%)	0 (0.0%)	1 (0.4%)		
Other	4 (1.4%)	1 (3.0%)	3 (1.2%)		
**Echocardiographic Parameters**					
LVEF (%)	64.89 ± 11.58	61.33 ± 12.21	65.34 ± 11.44	0.98 (0.95–1.01)	0.049
LVEF < 50%	19 (6.5%)	6 (18.2%)	13 (5.0%)	4.19 (1.47–11.92)	0.019
LVMI (g/m^2^)	138.20 ± 46.94	153.67 ± 47.74	136.22 ± 46.56	1.01 (1.01–1.02)	0.033
Maximal Wall Thickness (mm)	20.47 ± 4.59	20.64 ± 4.51	20.41 ± 4.60	1.02 (0.95–1.11)	0.558
MWT >/= 30	14 (4.8%)	1 (3.0%)	13 (5.0%)	0.59 (0.08–4.65)	0.497
LVOT gradient (mmHg) >/= 30	48 (16.5%)	6 (18.2%)	42 (16.3%)	1.14 (0.45–2.94)	0.808
More than Moderate MR	18 (6.2%)	5 (15.2%)	13 (5.0%)	3.37 (1.12–10.14)	0.040
LV E/e’	15.65 ± 8.40	20.64 ± 10.39	15.02 ± 7.92	1.08 (1.03–1.12)	<0.001
Elevated Filling Pressure	154 (52.9%)	24 (72.7%)	130 (50.4%)	2.63 (1.18–5.87)	0.462
LAVImax (mL/m^2^)	42.28 ± 20.92	61.75 ± 29.28	39.79 ± 18.11	1.04 (1.02–1.06)	0.001
LAVImin (mL/m^2^)	22.39 ± 16.70	38.26 ± 23.81	20.36 ± 14.41	1.05 (1.03–1.07)	<0.001
LAEF (%)	50.9 ± 14.60	40.5% ± 11.6%	52.2 ± 14.4	0.01 (0.01–0.06)	<0.001
**Strain Parameters**					
LASr ED	21.19 ± 12.11	11.12 ± 8.05	22.48 ± 11.95	0.89 (0.85–0.93)	<0.001
LASr ED <23	172 (59.1%)	31 (93.9%)	141 (54.7%)	12.86 (3.02–54.87)	<0.001
LAScd ED	−11.83 ± 8.20	−5.60 ± 4.29	−12.62 ± 8.24	1.25 (1.13–1.37)	<0.001
LAScd ED < 12	176 (60.5%)	31 (93.9%)	145 (56.2%)	12.08 (2.83–51.54)	<0.001
LASct ED	−9.33 ± 6.25	−5.31 ± 6.29	−9.85 ± 6.07	1.15 (1.07–1.23)	<0.001
LASct ED < 5	65 (22.3%)	16 (48.5%)	49 (19.0%)	4.01 (1.90–8.50)	<0.001
LVGLS	−12.92 ± 4.16	−9.47 ± 3.17	−13.36 ± 4.07	1.32 (1.17–1.47)	<0.001

ACE-I: angiotensin-converting enzyme inhibitor; ARB: angiotensin II receptor blocker; ARNI: angiotensin receptor/neprilysin inhibitor; LVEF: left ventricular ejection fraction; LVMI: left ventricular mass index; MWT: maximal wall thickness; LVOT: left ventricular outflow tract; E/e’: ratio of mitral inflow velocity to mitral annular velocity during early diastole; LAVI: left atrial volume indexed to body surface area; LAEF: left atrial ejection fraction; LASr ED: left atrial reservoir strain; LAScd ED: left atrial conduit strain; LASct ED: left atrial contractile strain; LVGLS: left ventricular global longitudinal strain.

**Table 2:** Characteristics of HCM patients in relation to composite adverse outcomes.

Variables	Overall (*n* = 291)	Heart Failure Hospitalization (*n* = 33)	Free from Heart Failure (*n* = 258)	Hazard Ratio (95% CI)	*p*-Value
**Baseline Demographics**					
Age (years)	58.75 ± 16.79	68.29 ± 15.72	53.91 ± 15.20	1.07 (1.05–1.09)	<0.001
Male, n (%)	209 (71.8%)	60 (61.2%)	149 (77.2%)	2.15 (1.27–3.63)	0.010
BMI	25.30 ± 4.48	24.99 ± 4.97	24.45 ± 4.21	0.98 (0.92–1.03)	0.404
Ethnicity				0.65 (0.45–1.29)	0.224
Chinese	194 (66.7%)	73 (74.5%)	121 (62.7%)		
Malay	53 (18.2%)	17 (17.3%)	36 (18.7%)		
Indian	35 (12.0%)	8 (8.2%)	27 (14.0%)		
Other	9 (3.1%)	0 (0.0%)	9 (4.7%)		
**Comorbidities**					
Hypertension	131 (45.0%)	61 (62.2%)	70 (36.3%)	2.90 (1.75–4.79)	0.001
Hyperlipidemia	117 (40.2%)	49 (50.0%)	68 (35.2%)	1.84 (1.12–3.01)	0.017
Diabetes Mellitus	47 (16.2%)	25 (25.5%)	22 (11.4%)	2.66 (1.41–5.02)	0.005
Ischemic Heart Disease	80 (27.5%)	36 (36.7%)	44 (22.8%)	1.97 (1.16–3.34)	0.013
Atrial Fibrillation	51 (17.5%)	24 (24.5%)	27 (14.0%)	1.99 (1.08–3.69)	0.034
Chronic Kidney Disease	3 (1.0%)	2 (2.0%)	1 (0.5%)	4.00 (0.37–44.67)	0.277
Previous Stroke	31 (10.7%)	22 (7.6%)	9 (4.7%)	5.92 (2.61–13.44)	<0.001
**Medication Use**					
Antiplatelet	87 (29.9%)	43 (43.9%)	44 (22.8%)	2.65 (1.57–4.46)	<0.001
Oral Anticoagulation	58 (20.0%)	29 (29.6%)	29 (15.0%)	2.48 (1.37–4.47)	0.003
Beta Blockers	189 (64.9%)	66 (67.3%)	123 (65.1%)	1.17 (0.70–1.96)	0.604
ACE-I/ARB/ARNI	82 (28.3%)	32 (32.7%)	50 (25.9%)	1.39 (0.82–2.36)	0.338
Calcium Channel Blockers	58 (19.9%)	27 (27.6%)	31 (16.1%)	1.99 (1.11–3.57)	0.045
Diuretics	49 (16.8%)	32 (65.3%)	17 (8.8%)	5.02 (2.61–9.64)	<0.001
Oral Antiarrhythmics	17 (5.8%)	8 (8.2%)	9 (4.7%)	1.82 (0.68–4.87)	0.201
**Type of HCM**					
Sigmoid Septum	52 (17.9%)	22 (22.4%)	30 (15.5%)	-	0.402
Reverse Curvature	44 (15.1%)	10 (10.2%)	34 (17.6%)		
Apical	105 (36.1%)	36 (36.7%)	69 (35.8%)		
Concentric	85 (29.2%)	28 (28.6%)	57 (29.5%)		
Mid Cavity	1 (0.3%)	0 (0.0%)	1 (0.5%)		
Other	4 (1.4%)	2 (2.0%)	2 (1.0%)		
**Echocardiographic Parameters**					
LVEF (%)	64.89 ± 11.58	63.44 ± 12.66	65.62 ±10.95	0.98 (0.96–1.01)	0.069
LVEF < 50%	20 (6.9%)	10 (10.2%)	9 (4.7%)	2.32 (0.91–5.92)	0.055
LVMI (g/m^2^)	138.20 ± 46.94	151.66 ± 52.30	131.36 ± 42.50	1.01 (1.01–1.02)	<0.001
Maximal Wall Thickness (mm)	20.47 ± 4.59	20.93 ± 4.76	20.24 ± 4.49	1.04 (0.98–1.09)	0.566
MWT >/= 30	14 (4.8%)	5 (5.1%)	9 (4.7%)	1.10 (.036–3.37)	0.571
LVOT Gradient (mmHg) >/= 30	48 (16.5%)	19 (19.4%)	29 (15.0%)	1.36 (0.72–2.57)	0.269
More than Moderate MR	18 (6.2%)	9 (9.2%)	9 (4.7%)	2.07 (0.79–5.39)	0.201
LV E/e’	15.65 ± 8.40	17.81 ± 9.91	14.57 ± 7.32	1.05 (1.01–1.08)	0.004
Elevated Filling Pressure	154 (52.9%)	65 (66.3%)	89 (46.1%)	2.30 (1.39–3.83)	0.001
LAVImax (mL/m^2^)	42.28 ± 20.92	49.47 ± 24.87	38.63 ± 17.57	1.02 (1.01–1.04)	0.024
LAVImin (mL/m^2^)	22.39 ± 16.70	28.28 ± 19.77	19.40 ± 14.03	1.03 (1.02–1.05)	<0.001
LAEF (%)	50.9 ± 14.60	46.6% ± 13.4%	53.2% ± 14.7%	0.05 (0.01–0.26)	<0.001
**Strain Parameters**					
LASr ED	21.19 ± 12.11	14.92 ± 10.11	24.37 ± 11.81	0.92 (0.89–0.95)	<0.001
LASr ED < 23	172 (59.1%)	77 (78.6%)	95 (49.2%)	3.78 (2.16–6.62)	<0.001
LAScd ED	−11.83 ± 8.20	−7.60 ± 5.83	−13.97 ± 8.40	1.16 (1.10–1.22)	<0.001
LAScd ED < 12	176 (60.5%)	83 (84.7%)	93 (48.2%)	5.95 (3.21–11.04)	<0.001
LASct ED	−9.33 ± 6.25	−7.25 ± 6.14	−10.39 ± 6.06	1.09 (1.04–1.14)	<0.001
LASct ED < 5	65 (22.3%)	35 (35.7%)	30 (15.5%)	3.02 (1.71–5.33)	<0.001
LVGLS	−12.92 ± 4.16	−11.02 ± 3.72	−13.88 ± 4.04	1.21 (1.13–1.29)	<0.001

ACE-I: angiotensin-converting enzyme inhibitor; ARB: angiotensin II receptor blocker; ARNI: angiotensin receptor/neprilysin inhibitor; LVEF: left ventricular ejection fraction; LVMI: left ventricular mass index; MWT: maximal wall thickness; LVOT: left ventricular outflow tract; E/e’: ratio of mitral inflow velocity to mitral annular velocity during early diastole; LAVI: left atrial volume indexed to body surface area; LAEF: left atrial ejection fraction; LASr ED: left atrial reservoir strain; LAScd ED: left atrial conduit strain; LASct ED: left atrial contractile strain; LVGLS: left ventricular global longitudinal strain.

**Table 3:** Multivariable analysis for left atrial parameters for HCM. baseline model comprising the following variables: age, sex, LV mass index, LVEF, presence of resting LVOTO, E/e’, and AF.

(a) Heart Failure Hospitalization			
Multivariable Models	Adjusted HR (95% CI)	*p*-value	C-statistic (95% CI)
Baseline Model + LAVImax	1.025 (1.006–1.043)	0.009	0.808 (0.738–0.879)
Baseline Model + LAVImin	1.030 (1.009–1.052)	0.005	0.818 (0.749–0.887)
Baseline Model + LAEF	0.068 (0.004–1.038)	0.053	0.816 (0.750–0.883)
Baseline Model + LACI	1.890 (1.020–3.883)	0.043	0.809 (0.739–0.878)
Baseline Model + LACI, cut-off > 40%	7.91 (2.871–11.784)	<0.001	0.854 (0.804–0.905)
Baseline Model + LASr ED	0.877 (0.829–0.928)	<0.001	0.861 (0.807–0.915)
Baseline Model + LASr ED, cut-off of 23	7.07 (1.55–32.67)	0.012	0.833 (0.776–0.889)
Baseline Model + LAScd ED	1.229 (1.106–1.366)	0.0001	0.844 (0.784–0.905)
Baseline Model + LAScd ED, cut-off of 12	6.95 (1.58–31.03)	0.010	0.833 (0.778–0.887)
Baseline Model + LASct ED	1.180 (1.086–1.281)	0.0001	0.838 (0.770–0.905)
Baseline Model + LASct ED, cut-off of 5	3.30 (1.59–6.88)	0.001	0.839 (0.781–0.896)
(b) Composite Adverse Outcomes			
Multivariable Models	Adjusted HR (95% CI)	*p*-value	C-statistic (95% CI)
Baseline Model + LAVImax	1.006 (0.995–1.018)	0.294	0.743 (0.691–0.796)
Baseline Model + LAVImin	1.011 (0.997–1.025)	0.139	0.746 (0.694–0.799)
Baseline Model + LAEF	0.469 (0.098–2.243)	0.343	0.744 (0.691–0.796)
Baseline Model + LACI	1.890 (1.020–3.883)	0.043	0.809 (0.739–0.878)
Baseline Model + LACI, cut-off > 40%	2.062 (1.323–3.213)	0.0014	0.761 (0.710–0.811)
Baseline Model + LASr ED	0.936 (0.912–0.961)	<0.0001	0.774 (0.725–0.824)
Baseline Model + LASr ED, cut-off of 23	2.01 (1.56–3.48)	0.013	0.752 (0.701–0.802)
Baseline Model + LAScd ED	1.110 (1.059–1.164)	<0.0001	0.766 (0.716–0.816)
Baseline Model + LAScd ED, cut-off of 12	3.31 (1.84–6.97)	<0.001	0.764 (0.715–0.812)
Baseline Model + LASct ED	1.083 (1.034–1.128)	0.0001	0.763 (0.711–0.814)
Baseline Model + LASct ED, cut-off of 5	2.47 (1.57–3.88)	<0.001	0.764 (0.712–0.915)

## Data Availability

The data presented in this study is available upon request from the corresponding author (due to privacy restrictions).

## Abbreviations

The following abbreviations are used in this manuscript:

HCM	Hypertrophic cardiomyopathy
VT	Ventricular tachycardia
VF	Ventricular fibrillation
ICD	Implantable cardioverter defibrillator
LA	Left atrial
LV	Left ventricular
EF	Ejection fraction
LVOT	Left ventricular outflow tract
GLS	Global longitudinal strain
HFpEF	Heart failure with preserved ejection fraction
LVMI	Left ventricular mass index
LASr	Left atrial reservoir strain
LAScd	Left atrial conduit strain
LASct	Left atrial contraction strain
LAVImax	Maximum left atrial volume indexed to body surface area
LAVImin	Minimum left atrial volume indexed to body surface area
SGLT2i	Sodium-glucose cotransport inhibitor
